# (S)Partners for Heart Health: a school-based program for enhancing physical activity and nutrition to promote cardiovascular health in 5^th ^grade students

**DOI:** 10.1186/1471-2458-8-420

**Published:** 2008-12-22

**Authors:** Joseph J Carlson, Joey C Eisenmann, Karin A Pfeiffer, Kathleen B Jager, Scott T Sehnert, Kimbo E Yee, Rita A Klavinski, Deborah L Feltz

**Affiliations:** 1 Department of Radiology, Division of Sports and Cardiovascular Nutrition, Michigan State University, East Lansing, MI, USA; 2 Department of Food Science and Human Nutrition Michigan State University, East Lansing, MI, USA; 3 Department of Kinesiology, Michigan State University, East Lansing, MI, USA; 4 Department of Pediatrics, Michigan State University, East Lansing, MI, USA; 5 College of Social Science, Department of Family and Child Ecology, Michigan State University, East Lansing, MI, USA; 6 Michigan State University Extension, Michigan State University, East Lansing, MI, USA

## Abstract

**Background:**

The American Heart Association Position Statement on Cardiovascular Health Promotion in Public Schools encourages school-based interventions for the primary prevention of cardiovascular disease (CVD) through risk factor prevention or reduction in children with an emphasis on creating an environment that promotes healthy food choices and physical activity (PA). In an effort to address issues related to CVD risk factors including obesity in Michigan children, a multi-disciplinary team of Michigan State University (MSU) faculty, clinicians, and health profession students was formed to "(S)partner" with elementary school physical education (PE) teachers and MSU Extension staff to develop and implement a cost-effective, sustainable program aimed at CVD risk factor prevention and management for 5^th ^grade students. This (S)partnership is intended to augment and improve the existing 5^th ^grade PE, health and nutrition curriculum by achieving the following aims: 1) improve the students' knowledge, attitudes and confidence about nutrition, PA and heart health; 2) increase the number of students achieving national recommendations for PA and nutrition; and 3) increase the number of students with a desirable CVD risk factor status based on national pediatric guidelines. Secondary aims include promoting school staff and parental support for heart health to help children achieve their goals and to provide experiential learning and service for MSU health profession students for academic credit.

**Methods/Design:**

This pilot effectiveness study was approved by the MSU IRB. At the beginning and the end of the school year students undergo a CVD risk factor assessment conducted by MSU medical students and graduate students. Key intervention components include eight lesson plans (conducted bi-monthly) designed to promote heart healthy nutrition and PA behaviors conducted by PE teachers with assistance from MSU undergraduate dietetic and kinesiology students (Spartners). The final 10 minutes of each lesson, MSU Spartners conduct small breakout/discussion groups with the 5^th ^grade students. Additionally, each Spartner case manages/mentors two to three 5^th ^grade students using a web-based goal setting and tracking protocol throughout the school year.

**Discussion:**

This paper describes the rationale, development, and methods of the Spartners for Heart Health program. This is a multi-level intervention designed to promote heart healthy behaviors and prevent or manage CVD risk factors in children. We believe this will be a viable sustainable intervention that can be disseminated and adopted by other institutions with minimal cost by engaging college students as an integral part of the measurement and intervention teams.

## Background

Cardiovascular disease (CVD) remains the leading cause of death in the United States (U.S.) [1]. Although the clinical manifestations of CVD tend not to occur until mid-adulthood, studies have shown that atherosclerosis has its origins in childhood and adolescence [2-6]. There is also evidence that CVD risk factors track from childhood/adolescence into adulthood and predict CVD morbidity [7]. Due to the early origins of atherosclerosis and the increasing prevalence of pediatric obesity and other CVD risk factors, there is considerable interest in the cardiovascular and metabolic health of youth [8].

The current obesity and CVD risk factor problem among U.S. youth is in part linked to physical inactivity and poor dietary habits. Approximately 40% of U.S. children 6–11 years of age meet current physical activity recommendations [9] and trends in dietary intake suggest a higher energy density and lower nutrient density. For instance, intakes of soda and fruit drinks account for between 15–20% of children's total caloric intake, while less than 20% of youth achieve the recommendation of five or more fruits and vegetables per day and less than 15% of youth consume two or more servings of whole grains per day [10-12]. Indices of diet quality (e.g., the healthy eating index) also reflect a low nutrient-dense dietary pattern [13]. These dietary and physical activity patterns are of particular concern among children living in low socio-economic status (SES) households, placing them at an even greater risk of physical inactivity, poor diet, and CVD [14,15]. Taken together, this evidence highlights the importance of promoting healthful dietary and physical activity behaviors during childhood and adolescence, particularly in low SES populations.

With respect to prevention of CVD, the public school setting is frequently targeted for intervention programs because it reaches large segments of youth from all SES groups [16-19]. Guidelines from the Centers for Disease Control and Prevention have specifically emphasized the importance of school programming for modifying physical activity [16] and dietary behaviors [20] of children. The American Heart Association (AHA) position statement on Cardiovascular Health Promotion in the Public Schools also states that the primary prevention of CVD in childhood via changes in dietary and physical activity habits are important [1,21]. The aforementioned epidemiological and clinical evidence was also the premise for an additional AHA statement regarding the Prevention of Atherosclerotic CVD Beginning in Childhood [22]. These guidelines also emphasize the importance of diet and physical activity for the prevention or management of CVD risk factors to reduce morbidity and premature mortality in adults [22]. The guidelines point out that one-third of the 2010 national health objectives can be significantly influenced by school health programs [1,23], and thus encourage school interventions for heart health promotion and CVD risk reduction for children with an emphasis on creating an environment that promotes healthy food choices and physical activity.

Overall, previous school-based studies have produced moderate effectiveness for the adoption of healthy lifestyle behaviors. However, most of these studies had some significant limitations [24]. A common example is that the focus of the physical activity and dietary behaviors in which children engage is the school day, with minimal emphasis on what kids do outside of school. In most cases, this relates to the involvement of the family and/or community. In particular, most school-based studies either neglect to address or poorly address the importance of family support [24], which has been identified as an important factor in both the adoption and maintenance of behavior change in children [25,26]. Furthermore, evidence for the diffusion and maintenance of physical activity has been lacking in many intervention studies [24] with the exception of "CATCH" [27,28] and "SPARK" [29], which both showed positive results. In an effort to address the aforementioned limitations, some researchers have suggested that multi-level interventions that aim to influence healthy lifestyle behaviors both at school and outside of school by including the family may prove more successful [30,24,26,33].

This paper presents a synopsis of "(S)partners for Heart Health", a multi-level intervention aimed at promoting healthful nutrition and physical activity behaviors related to cardiovascular health and CVD risk reduction. The format of this paper consists of an overview of the project including the project aims, development steps for the intervention and measurement protocol, and a detailed description of the protocol that will be implemented in an effectiveness study. The paper concludes with a discussion of the implications and future plans of the intervention.

### Study Protocol Overview

*What is (S)partners for Heart Health? *(S)partners for Heart-Health was conceptualized and designed by a multi-disciplinary team of Michigan State University (MSU) faculty (with backgrounds in nutrition, exercise physiology, physical education, child growth and maturation, psychology, and public health), MSU health clinicians, medical students and allied health profession students and MSU Extension staff. The main objective for the multi-disciplinary team is to "(S)partner" with elementary school physical education teachers and university extension staff to develop and implement a cost-effective sustainable intervention program for CVD risk factor prevention among 5^th ^grade students. To "(S)partner" refers to forming a partnership between students, faculty, and staff of public schools and MSU, where the mascot is a Spartan. The (S)partnership is intended to augment the existing 5^th ^grade physical education, nutrition and health curriculum to sustain or improve heart-healthy behaviors and health status in the 5^th ^grade students by achieving the following primary and secondary aims:

#### Primary aims

1. To increase the percentage of students achieving national recommendations for physical activity and nutrition behaviors;

2. To improve the public school students' knowledge, attitudes and self-efficacy about heart healthy nutrition and physical activity behaviors as recommended by national guidelines;

3. To improve or maintain the number of students with a desirable CVD risk factor status;

#### Secondary aims

1. To promote school staff and parental support for heart healthy activities to help children achieve their heart health goals;

2. To provide applied hands-on learning and training for MSU health profession students.

There are several unique aspects of the (S)partners for Heart Health program that are designed to address the limitations of previous school-based cardiovascular health trials. The project is designed to be cost-effective and sustainable by utilizing undergraduate dietetic and kinesiology students and second year medical students to play key roles in the program intervention and measurement assessment, respectively. The kinesiology and dietetics students will (S)partner with 5^th ^grade students by mentoring and case managing the students using a secure (password protected) web-based goal setting and tracking protocol and through involvement in monthly lesson plans and small breakout meetings. The medical students will carry out the measurement protocol.

#### Theoretical basis of (S)partners for Heart Health

The theoretical basis incorporates components of Bandura's Social Cognitive Theory [34] to promote the students' self-efficacy for making positive nutrition and physical activity choices that ultimately will maintain or promote a desirable CVD risk factor status. Additionally, we have incorporated and adapted facets of goal setting and group education methodology previously used with adults [35-37], given limited research on goal setting for health behavior change with children [33,38]. Furthermore, we will encourage and facilitate parental awareness and community support for the program goals which can potentially influence not only the school environment, but also the surrounding community.

### Development and Pilot Phase

In preparation for the effectiveness study, we conducted a formative assessment, evaluated and selected curricula, recruited and prepared students for work with the assessment and intervention, and conducted a pilot study of each component of the measurement and intervention protocol. The following summarizes the primary steps of the development and pilot phase.

#### Formative assessment

In order to inform the investigators regarding best practice for the impending intervention protocol, focus groups were conducted with local stakeholders (one group each of 5^th ^grade students, their parents, and teachers and administrators) in the community of interest. Each group was facilitated separately by a trained qualitative researcher, who posed a framework of questions related to the (S)partners program. The assortment of empirical material generated by the focus groups enhanced participant control and ownership of the research, as well as provided a more naturalistic setting in which participants could validate and discuss experiences and viewpoints of similar others[39]. The focus group with students included gathering visual empirical materials; the researchers adapted the Kinetic Family Drawing Test [40] to elicit an additional form of expression as it relates to the richness and complexity of the students' focus group dynamics.

All focus groups were audio-taped while a research assistant recorded field notes. Each group dialogue was transcribed by a trained research assistant who was informed about the focus group experience and had participated in the decision-making rules for transcription [41]. Content and thematic analysis was adapted for focus group methods and occurred in data-specific phases that identified themes linked to individual perceptions, group narratives and within- and between-group similarities and/or differences[39]. Local conditions were compared with existing public knowledge to identify CVD prevention themes while informing development and refinement of the (S)partners curriculum[39].

#### Evaluation of curricula

The investigative team thoroughly examined the literature regarding existing age-appropriate curricula for promoting positive physical activity and nutrition behaviors. After obtaining copies of the curricula and systematically evaluating available options, "Jump Into Foods and Fitness" (JIFF) was selected for this intervention [42]. The curriculum can be used by public school physical education teachers and includes both physical activity and nutrition material. The curriculum was selected because it meets the intervention objectives as well as the requirements for the Michigan Department of Education. In addition to matching the goals of this project, it is age-appropriate for 5^th ^graders. The curriculum also offers an opportunity for college students to be actively involved in the lesson plans. Importantly, JIFF was developed in conjunction with MSU Extension by experts in physical education and nutrition programming for children making it compatible with existing community-based efforts led by MSU Extension representatives across the state of Michigan.

#### Kinesiology and Dietetic student recruitment and preparation

The research team used email solicitation followed by informational presentations and question and answer periods to recruit MSU students to participate. To qualify, students needed to be a junior with a 3.0 grade point average or higher and had to agree to be involved for three semesters (spring of Junior year and both fall and spring semester of their senior year) and enroll for one credit each semester. Members of the investigative team developed the training curricula for the kinesiology and dietetic students to prepare them to (S)partner with the 5^th ^grade students. Approval was gained from both the kinesiology and dietetics departments to offer this experiential learning for credit. The training class included readings, lectures, discussions, web-based assignments, small group assignments and discussions (Table 1).

**Table 1:** Summary of training topics for dietetic, kinesiology, and medical students in the (S)Partners for Heart Health program.

**Undergraduate dietetic and kinesiology student education and training**	**Medical student education and training**
Pediatric guidelines and recommendations for nutrition and physical activity from the American Heart Association and the American Academy of Pediatrics	Pediatric guidelines and recommendations for nutrition and physical activity from the American Heart Association and the American Academy of Pediatrics, the research protocol, and content on conducting valid and reliable measures.
	
Completion of the University Institutional Review Board (IRB) training	Completion of the MSU IRB and Blood borne pathogen training
Fundamentals of goal setting and principles and applications of Albert Bandura's theory of self-efficacy for use in goal achievement	Completion of the MSU Blood borne pathogen training
	
A review of CVD risk factors and the role of nutrition and physical activity in preventing and managing CVD risk factors.	Knowledge and techniques for interacting with 5^th ^graders including sensitivity issues
	
Tips for communicating with 5^th ^graders including sensitivity issues	Training for performing reliable and valid of physical measures and CVD risk factors
	
"Mock" web-based goal setting and tracking with one MSU student role playing as 5^th ^grade students and the other serving as the MSU (S)partner	
	
"Mock" break-out meetings in small groups (4–5 per group) with half of the MSU students role playing as 5^th ^grade students and the other serving as the MSU (S)partners	

#### Medical student recruitment and preparation

A group of first year medical students were identified from a pool of students who were seeking to get involved with a community-based project that included children and health. These students then recruited other students within their class. The training included selected readings and meetings with the project investigators to learn about the project aims, the measurement battery basis, and hands-on practice sessions to perform the measurement battery (Table 1).

#### Pilot study

The final development activity was conducting a pilot study. The purpose of conducting the pilot study was to prepare for the larger-scale effectiveness trial. The goals of the pilot testing were to fine-tune logistics for conducting the measurement protocol, practice delivering the intervention components (lesson plans, small breakout meetings and web-based goal setting and tracking), and develop a process evaluation plan for assessing implementation of the intervention components. The pilot testing occurred during a three-month interval in a public school in a small rural town in central Michigan. The pilot study was initiated following parental consent and assent from 5^th ^grade students who agreed to participate in the pilot study.

**Figure 1:**
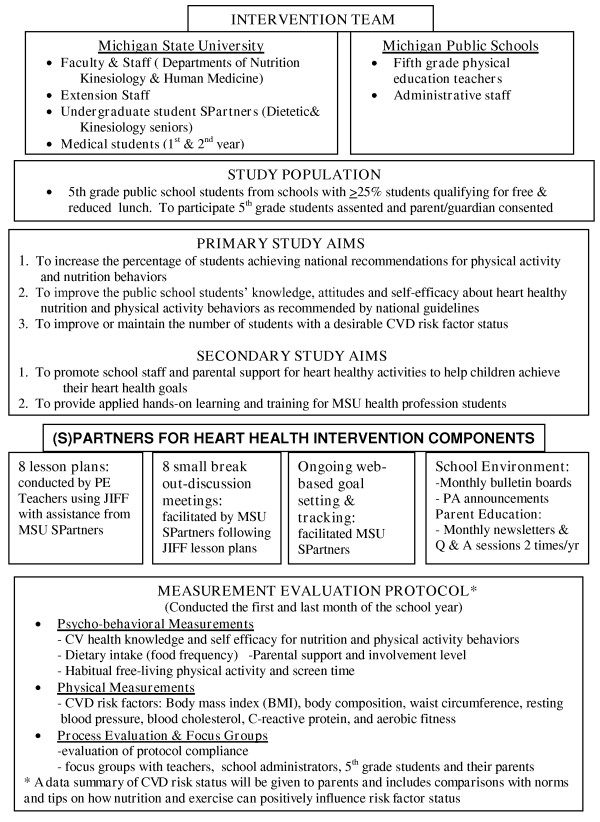
**Summary of the (S)Partners for Heart Health intervention team, study population, study aims, intervention components and measurement protocol**.

Based on the pilot experience, and knowledge gained from current and past literature, the investigators finalized the intervention and measurement battery for the effectiveness study to be conducted in multiple schools over an entire school year. The intervention and measurement evaluation components are summarized in Figure 1.

## Methods/Design

### Effectiveness study population and recruitment

This parallel design intervention trial has been approved by the MSU Institutional Review Board. Also approval from each of the participating school's board of education was also obtained.

Inclusion criteria for the effectiveness study includes 5^th ^grade classrooms within 50 miles of MSU with 25% or more of students qualifying for free or reduced lunch to ensure a portion of the population are at an increased risk of health disparities. Four schools will participate in the effectiveness trial. Two physical education classrooms (one boys and one girls) within each school will be included for a total of eight physical education classes. Two of the participating schools will be assigned to the (S)partners for Heart Health protocol and the other two will be assigned as the usual care comparison school. Depending on the school, physical education class size will vary from 25–35 students. We anticipate the total number of students will be approximately 180–200.

### Intervention protocol

The overall intervention protocol includes lesson plans facilitated by the physical education teacher in addition to other key components facilitated by the Dietetic and Kinesiology students who will case manage [or (S)partner] the 5^th ^grade students. Following the completion of the lesson plans, the MSU (S)partners will conduct small group breakout meetings and on-going goal setting and evaluation via a web-based goal tracking and education program. A detailed description of each of these components is provided below.

#### Lesson plans

The eight JIFF lesson plans will be presented during the fall semester following the completion of the baseline measurement (mid-October) and will be taught by the school physical education (PE) teachers with assistance from the (S)partners. As noted previously, the "Jump into Foods and Fitness" (JIFF) curriculum provides the foundation of the eight lesson plans. Prior to the first JIFF lesson there will be an introductory lesson that describes the (S)partners program and includes content on the role of nutrition and exercise in heart health (CVD risk factors) and preventing cardiovascular disease. Prior to the school year, PE teachers will be provided with copies of the curriculum and undergo a two-hour training session with a trained PE teacher who is a member of the investigative team. Training will include guidelines for implementation, including guidance on how to use the curriculum with an ideal match for their particular environment as long as they ensure that they attempt to meet program goals. Meeting goals includes having children be engaged in moderate-to-vigorous physical activity for at least 50% of class time, providing activities and opportunities that help children enjoy PE, providing verbal prompts to be active, and participating in activity with the children. To augment the training and to help standardize the implementation of JIFF and meeting of goals, all teachers will receive an instruction list for using JIFF. Additionally, PE teachers will be asked to document any modifications they make to the curriculum, and the process evaluation procedures (see Process Evaluation section) will account for the changes.

#### Case management and mentoring [(S)partnering]

Mentoring and tutoring programs are widely used in non-profit organizations including universities and public schools for a variety of goals including educational, life skills, and health habits [43,44]. There is limited research evaluating the effects of these programs influence on health habits and health status of children however, a recent study demonstrated that 4^th^-7^th ^graders trained by their teachers to be peer educators for kindergarten through 3^rd ^grade students, show positive effects on health habits and health status as compared to controls [44]. Additionally, peer mentoring programs can be cost effective [43], offer benefits to the students being mentored, and also benefit those students conducting the mentoring or tutoring [44-46]. In this project, the (S)partners will mentor/tutor the 5^th ^grade students to assist them in learning about the role of nutrition and physical activity in preventing or managing CVD risk factors for their health. This will be achieved by a combination of monthly participation in classroom activities under the direction of the schools' physical education teachers and MSU staff, as well as weekly internet communication designed to facilitate the 5^th ^grade students' achievement of suggested nutrition and physical activity behavioral goals (all internet communication are subject to MSU staff oversight). The goal is for each (S)partner to mentor/case manage 3–4 fifth grade students during the school year.

To help make this intervention sustainable and cost-effective, we have developed an independent study in which kinesiology and dietetic students enroll for three semesters. The first semester (Spring) involves training to be an active (S)partner for the fall and spring semesters of the following year. This component was developed specifically to help achieve our project goals using principles from previous research [43-46] and modified based on feedback from kinesiology and dietetic students who participated in the training course, and focus groups during our pilot study (Fall 2007-Spring 2008). The Kinesiology and Dietetic students (S)partners are trained to help facilitate the achievement of the 5^th ^grade heart- healthy behavioral goals during the school year. The (S)partners will communicate with the students for whom they are responsible on a weekly basis using a web-based goal tracking and education protocol. On a monthly basis the (S)partners will participate in physical education lesson plans which is followed by small breakout meetings that includes three or more (S)partners meeting with 8–10 fifth grade students for 10–15 minutes. The goal of these meetings are to answer questions related to the lesson plans and to discuss successes and barriers related to the achievement of their nutrition and activity goals.

#### Web- based goal tracking and education

In the time between monthly visits to the public schools for lesson plans and small group meetings, (S)partners will communicate with the school children via a secure website  which is monitored by study coordinators graduate students and faculty. This web-based platform allows for the facilitation of behavioral goal setting for nutrition and physical activity using a behavioral tracking tool, as well as links to educational modules to promote the achievement of heart healthy behavioral and educational goals and reinforce classroom educational efforts. Our protocol is developed based on previous relevant research involving goal setting [35,36,47-50] and knowledge gained from the pilot study. Students are required to log on weekly at school and set their activity and nutrition goals for the week, which will be measurable and based on national guidelines for nutrition and physical activity [51-54]. Students will maintain the same goal for a minimum of three weeks or until they achieve it. For example, a student who reports a low intake of whole grains (1 serving per day) may set a goal to have 3 or more servings of whole grains per day. Success will be based on achieving the goal at least 5 of 7 days per week. The students will log their progress for their nutrition and physical activity goals on tracking cards and then they will log their progress weekly on the (S)partner website. The (S)partners will give guidance to help them achieve the goal. To avoid barriers to computer access, there will be a set time weekly at the school during which the 5^th ^grade students report on their goals and communicate with their (S)partner. If children have access at home, they will be encouraged to access the website more often.

#### School Support

To promote healthful lifestyle behaviors among the school staff and promote support for the (S)Partners for Heart Health program, school staff will receive monthly newsletters and will be invited to attend an evening meeting which summarizes the (S)partner activities and provides tips for promoting nutrition and physical activity at school two times per year (once in the fall and once in the Spring). Additionally, school staff will be given access to the (S)partner website that will include links and materials they can access. The school principal will read nutrition and exercise promotion tips over the school intercom weekly and an informational (S)partners for Heart-Health bulletin board will be posted monthly in a central location in the school throughout the year.

#### Parental support and involvement

To promote parental understanding and support of (S)partners for Heart Health, parents of the 5^th ^grade students will receive monthly newsletters including encouragement to access the (S)partners for Heart Health website that will include links and materials they can access. They will receive a copy of their child's CVD risk factor profile immediately following baseline measurement with specific tips to help their children sustain or achieve healthful, physical activity and nutrition patterns. They will also be invited to attend the evening meetings (one in the fall and one in the spring) to summarize the (S)partner activities and provide tips for promoting nutrition and physical activity outside of school.

#### Measurement protocol

Assessments will be conducted during September (pre-intervention) and May (post-intervention) by MSU medical students and staff. This service is part of the medical students' community outreach elective and is an ongoing elective that will promote cost-effective, long-term sustainability of the study protocol. The assessment includes physical activity and nutrition behaviors; lifestyle and heart health knowledge, attitudes and behaviors for nutrition and physical activity; and self-efficacy toward nutrition and exercise behavioral goals. The CVD risk appraisal includes resting blood pressure, blood cholesterol, C-reactive protein, body size and composition, and aerobic fitness. The baseline assessment data will help guide the heart healthy nutrition and exercise goal setting mentioned in the previous section.

#### Knowledge, attitude, behavior and self-efficacy evaluations

A battery of surveys that includes questions about heart health knowledge, attitudes and self-efficacy toward lifestyle will be completed by the child. The nutrition, exercise, and heart health knowledge and attitude tools include materials and methods used in previous heart health research in children and the surveys included in the JIFF manual [42,55,56]. Self efficacy for nutrition, exercise and heart health behaviors will be assessed using a 5-point scale appropriate for children that uses circles from small to large reflecting level of confidence [57,58].

#### Habitual free-living physical activity

Habitual, free-living physical activity will be assessed by a pedometer (Digiwalker 200-SW) over a 1-week period. The subjects will be given instructions for wearing the pedometer during the school day. Prior to wearing their assigned pedometers, students will check them using the shaker test and the 10-step test. Pedometers that do not function properly during these 'validity' checks will be replaced. Participants will record the time on/time off and number of steps accumulated on a customized index card. Subjects will also record comments related to activities performed while not wearing the pedometer and other comments regarding any compliance-related issues. To be included in the statistical analysis, participants must wear the pedometer at least 4 days (3 week day and 1 weekend) for at least 10 hours. Data will be expressed as both the number of steps per day and percentage of days for which national recommendations for the number of steps per day (13,000 and 11,000 steps per day for boys and girls, respectively) are achieved [59-61]. The weekly amount of time viewing television, playing video games, and online computer use will be self-reported by the child. Children will also be asked to indicate the number of hours they watch/play on weekdays and weekends for each of the three screen media. The percentage of students viewing ≤ 2 hours per day of screen time [62] will be determined.

#### Dietary intake

Dietary intake will be assessed using the Block Kids Food Frequency Questionnaire for Kids (Block Dietary Data Systems, Berkeley, CA; ). Program staff will be trained to administer the survey per company instructions in a standardized manner. The administration will take place in a class room group setting. Following completion of the questionnaires they will reviewed by staff for completeness and mailed back to Nutriquest for analysis. The report includes daily estimates of macro-nutrients, major micro-nutrients, and servings per day of key food groups. It is established that fifth grade ages are a difficult age group to measure dietary intake in. A couple of recent studies have evaluated the validity of the Block Kids Food Frequency Questionnaire using age groups similar to our study population's ages. When comparing a single 24-hour recalls to the Block Questionnaire modest interclass correlations (≥ 0.30) were revealed for key nutrients, except for protein and fruit and vegetable servings [63]. However in another study that used three day food logs (versus a single 24-hour recall) for estimates of 100% juice, milk, calcium, and vitamin D intakes the correlations were higher (r = 0.51–0.55) [64].

#### Cardiovascular disease risk factors

As previously indicated, the CVD risk factor profile will include: body mass index (BMI), body fat, waist circumference, resting blood pressure, blood cholesterol, C-reactive protein, and aerobic fitness. Obesity indicators will include measurements of body size and composition. The BMI will be calculated from measured stature and body mass according to standardized procedures. Waist circumference will be measured using a Gullick Anthropometric Tape at the level of the umbilicus. For anthropometric indices, the medical students will be trained prior to data collection by the investigators, and intra- and inter-observer measurement error will be determined. This type of training protocol has resulted in small measurement errors during the actual measurement period (SEM = 0.3 cm standing height; 0.1 kg body mass; 0.2 cm WC). Body fatness will be estimated using a foot-to-foot bioelectric impedance device (Tanita Corporation, Tokyo, Japan). Resting blood pressure will assessed using manual blood pressure device with the appropriate sized cuff on the subject's upper right arm following standardized procedures recommended by the Joint National Committee (USA) on Prevention, Diagnosis and Management of Hypertension [65]. Blood samples will be obtained by fingerprick and collected in heparinized capillary tubes. Blood sampling by fingerprick has been chosen for reasons of compliance and feasibility. Compared to venipuncture, blood sampling by fingerprick is less invasive and is less likely to deter subjects from participating; fingerprick is also more feasible for a field-based setting. One sample will be analyzed for total cholesterol, high-density lipoprotein-cholesterol, and triglycerides within 5 minutes by a portable cholesterol/glucose analyzer according to the protocol of the manufacturer (Cholestech LDX System, Hayward, CA). A second sample will be analyzed for C-reactive protein also using the Cholestech LDX. Cardiorespiratory fitness will be estimated using the PACER test which consists of an endurance-type shuttle run which will be conducted by program staff trained to conduct this procedure following standardized procedures [66].

#### Process evaluation (protocol compliance)

Protocol compliance will be conducted throughout the study to determine how dose delivered and fidelity to each component of the intervention relate to 5^th ^grade student outcomes. Physical education teachers will use checklists to keep track of when they are conducting lessons, and the project coordinator will randomly observe selected lessons at each intervention school. In addition, teachers will be asked to record any modifications and/or feedback they have regarding lessons and will report this information to project staff during interviews (post-intervention). Checklists and observations will be used to assess changes related to school environment (bulletin board, principal announcement, newsletters, and meetings). Physical education teachers and principals will also be queried about school environment during interviews (post-intervention); this includes documenting secular changes at control schools via interviews. MSU student (S)Partners will submit goal sheets created with the 5^th ^grade students. Attendance will be taken at each activity requiring (S)Partner presence, and each (S)Partner will complete a survey regarding his/her experiences (midpoint and post-intervention). Project staff will monitor log-ins to the website and content of (S)Partner/5^th ^grade student interactions. Finally, 5^th ^grade students will report their perceptions of the JIFF lessons, the web-based reporting tool, and experiences with (S)partners.

#### Statistical Power Calculations

A power calculation indicated that based on an N of 180 and 90 children in each group (SPartners for Heart Health versus comparison group), the following changes will need to be achieved for the primary aims of this study. With respect to the percentage of students achieving the national recommendation for physical activity; based on an estimate of 50% of the students achieving physical activity guidelines at baseline, it will be possible to detect a difference of 18% in the percentage achieving the guidelines (an increase from 50% to 59%) with an alpha level of 0.05 and a power of 80% using a one-tailed test. For nutrition behavior we chose total dietary fiber intake since it is surrogate marker of fruit, vegetable, whole grain and legume intake. Based on an estimated baseline mean intake of 12 ± 8.8 grams of dietary fiber per day, it will be possible to detect a difference of 3.3 grams of fiber per day with an alpha level of 0.05 and 80% power using a one-tailed test.

## Discussion

Following the completion of the effectiveness study described here, we intend to conduct a large-scale effectiveness trial with an adequate number of schools necessary for conducting school level statistical analysis (e.g., linear mixed models). We will develop partnerships with other colleges and universities which will be instrumental in the long-term goal of disseminating the (S)partner for Heart Health intervention in other regions of Michigan. In addition to partnering with other colleges and universities in Michigan, we plan to expand our relationship with MSU Extension through the agencies located in all counties in Michigan. In addition to the investigative team, the extension staff will be involved in supporting the training of the college students and assisting public school teachers in implementing the interventions. For example, Central Michigan University (CMU) could have a program based on their mascot called "Chippewas for Heart Health." In collaboration with the MSU investigative team, CMU faculty, staff, students, and MSU Extension staff would implement a program in counties surrounding Mount Pleasant, Michigan. This model (if determined to be effective) could feasibly be sustained long-term with modest financial support due to several reasons. The measurement and intervention battery (including the web-based tracking) could be adopted by any college and university that has allied health profession students and/or medical or nursing students. The fact that the key personnel conducting the measurement and the intervention consists of students receiving academic credit for this experience creates an ongoing flow of personnel and limits the costs associated with employing a research staff. Additionally, a partnership with MSU Extension will also help this program model be sustainable. Lastly, the measurement battery of tests could be reduced and the focus would be on monitoring behavior change.

Despite the many strengths of the intervention, there are some potential limitations. To date, we have only conducted a small pilot and feasibility study to prepare for the upcoming effectiveness study. Although formative assessment and the pilot have been performed, the goal of the upcoming investigation is to gain further information that will assist with a larger-scale implementation of the intervention. Thus, given the small number of schools that will be involved in the effectiveness study, it will not be possible to perform school-level analyses. In addition, schools were not randomized and blinding is impossible. Furthermore, over a 9-month period it will be a challenge to observe significant between group changes when comparing the (S)partner intervention with control schools. Other potential limitations that are difficult to anticipate but will be documented are concurrent health initiatives that may begin in a community during the effectiveness study. Finally, the number of (S)partners and medical students available and willing to be involved with this project during any given semester could be limiting to the number of schools and public school students that are served.

In summary, we have described the background and rationale, study design, measurement procedures, intervention components, and process evaluation procedures for (S)Partners for Heart Health. We anticipate this intervention will lead to an effective heart healthy education and behavior change model that can be cost effectively adopted into public schools to promote a desirable CVD risk factor status in school children. This will be achieved in part by using existing resources (MSU Extension) and activating college students (medical students and allied health professional students) in service learning through independent- and elective- academic credit, that offer training and experiential learning that cannot be achieved in the classroom setting. Although we are currently implementing this program in 5^th ^grade classrooms, the same methods can be applied to other grades with minor modifications (e.g., selection of different curricula). Ultimately, this program aims to reduce premature morbidity (obesity, hyperlipidemia, hypertension, diabetes, etc.) and mortality and their associated health costs by sustaining or improving heart healthy behaviors that influence CVD risk factor status in 5^th ^grades students acutely and long-term.

## Competing interests

The authors declare that they have no competing interests.

## Authors' contributions

JJC conceptualized the key components of the (S)Partners for Heart Health intervention; JJC, KAP, JCE designed the study; JJC, JCE, KAP and DLF established methods, measures, questionnaires; RAK, STS, and KEY assisted in the literature review and study coordination, and training MSU dietetic and kinesiology students. DLF and KAP provided insight on the conceptual, theoretical framework of the study. KAP devised the process evaluation. KBJ, KAP, RAK and JJC contributed to the development of the focus groups. KEY summarized focus group audio tapes to text and KBJ processed and summarize the focus group data. All authors participated in the writing of the paper and provided comments on the drafts and approved the final version.

## Pre-publication history

The pre-publication history for this paper can be accessed here:


